# Probiotic Supplementation Improves the Clinical Measures of Cognition in Patients With Mild Cognitive Impairment

**DOI:** 10.7759/cureus.89302

**Published:** 2025-08-03

**Authors:** Annabelle Assad, Samuel L Johnson, Rhett A Reichard

**Affiliations:** 1 Medicine, Saba University School of Medicine, The Bottom, BES; 2 Medical Education and Simulation, Saba University School of Medicine, The Bottom, BES; 3 Medical Education, Western Atlantic University School of Medicine, Freeport, BHS

**Keywords:** bifidobacterium, cognitive dysfunction, gut microbiome, lactobacillus, mild cognitive impairment, neuroinflammation, probiotics

## Abstract

Mild cognitive impairment progresses slowly and may be reversible, providing a window of opportunity for intervention before it progresses to Alzheimer's disease, at which point treatments, at best, ameliorate symptoms with little efficacy towards delaying disease progression. The gut and brain communicate through the gut-brain axis, and derangement of the gut microbiome has been shown to promote neuroinflammation, a process intricately linked to pathological progression to mild cognitive impairment and subsequent neurocognitive diseases. In preclinical trials, probiotics modulated the gut microbiome in a way that was neuroprotective. We aim to test our hypothesis that probiotic supplementation can improve cognition in those with mild cognitive impairment. A literature search of electronic databases PubMed/MEDLINE using terms such as "Probiotics" and "Cognitive dysfunction" or "Alzheimer's disease" or "Mild Cognitive Impairment" was done to identify all randomized controlled trials that tested our hypothesis. The effects of daily doses of *Bifidobacterium*, *Lactobacillus*, or a mixture of both on clinical cognitive tests were assessed in five trials. All studies but one demonstrated a statistically significant improvement in total cognitive test scores, while all studies showed a significant improvement over the placebo in at least one cognitive subdomain. An overall trend suggested greater improvement in those more severely impaired at baseline. Thus, probiotics may be effective in improving cognition in those with mild cognitive impairment. However, larger-scale multicenter clinical trials should confirm the present findings using a standardized approach.

## Introduction and background

The increasingly aging global population over the past several decades necessitates vigilant development and refinement of efficacious treatment for age-related neurocognitive disorders and pathologies. As neurodegenerative diseases progress, the likelihood of full recovery greatly diminishes due to irreversible neuronal damage, resulting in a bleak prognosis culminating in death [1,2]. Many neurodegenerative diseases, including Alzheimer's dementia (AD), initially present as mild cognitive impairment (MCI) with the likelihood and rates of progression variably reported [3-6]. The canonical definition of MCI broadly describes a transitional cognitive stage intervening normal decline associated with aging and neurodegenerative decline [3]. Currently, MCI is being clinically defined as a reported change in cognition with impairment in at least one clinical cognitive domain, objectivated by a below normative performance on a validated neuropsychological test (1-2 standard deviations below expectations depending on the criteria used), preserved capacity for independence in activities of daily living, and, finally, no evidence of dementia [7-10].

Although comprehensive neuropsychological assessments are considered the gold standard for diagnosis, current criteria do not specify which assessment tool should be used to operationalize MCI, allowing great flexibility in the choice of test [11,12]. Examples of comprehensive assessments include the Repeatable Battery for the Assessment of Neuropsychological Status (RBANS), the Consortium to Establish a Registry for Alzheimer's Disease (CERAD) neuropsychological assessment battery, and the Computerized Neurocognitive Function Test (CNT) batteries [13,14]. Diagnosing using a brief cognitive screening tool or based on subjective complaints solely should be avoided, as they may, on their own, fail to catch the subtle deficits associated with MCI. Examples of screening tests include the Mini-Mental State Examination (MMSE), the Montreal Cognitive Assessment (MoCA), the Alzheimer's Disease Assessment Scale-Cognitive subscale (ADAS-Cog), and the Mild Cognitive Impairment Screen (MCIS) [15-18].

MCI can be further classified as amnestic or non-amnestic. The different subtypes of MCI reflect distinct neurocircuitry that subserves distinct domains of cognition [19]. Because screening tests cannot distinguish between domains impaired, it is wise to assess all cognitive domains relevant to MCI irrespective of subtype, i.e., focusing on various aspects of memory, as well as attention and executive functions, having displayed a stable pattern of deficits and being recognized as early indicators of transition from MCI to AD [20]. 

Current data shows that a third of those with MCI will progress to AD within five years. Fortunately, recent studies have suggested that not all will show additional decline and that up to a quarter may even show reversal of the condition [21]. Given this possibility, it seems pertinent to explore therapeutic alternatives to prevent progression or promote reversal of the condition before it reaches more detrimental stages, at which point treatments, at best, ameliorate symptoms with little efficacy towards delaying disease progression [22,23].

A complex orchestration of underlying mechanisms associated with the onset, progression, and transition to pathological states of age-related cognitive decline has been modeled extensively. Neuroinflammation and associated microglial activation have been intricately linked to exacerbated cognitive decline, as well as pathological progression to MCI and subsequent neurodegenerative diseases [24-27]. Additionally, neuroinflammation can result from not only local insults, including compromised cerebral blood supply and microglial activation, but also systemic inflammation [28-37]. Accordingly, prophylactic and therapeutic treatment of MCI must address both neural and systemic correlates of neuroinflammation.

It is now well-established that the gut and brain communicate and modulate each other through the gut-brain axis and that both derangement of the gut microbiome and gut inflammation compromise the blood-brain barrier (BBB), contributing to neuroinflammation, neuronal death, and ultimately AD [38-40]. In fact, intestinal dysbiosis, defined as a microbial imbalance caused by an overgrowth of "bad" bacteria inside the gut, is thought to result in the decreased production of essential metabolites and/or the synthesis of harmful ones [41-43]. For instance, several studies have shown that "bad" bacteria populating the gut can produce lipopolysaccharides (LPS), known immunogenic constituents of beta-amyloid plaques, suggesting that bacterial parts can disrupt gut barrier integrity and travel from the gut to the brain to exacerbate Ab deposition [44,45]. Accumulation of Ab plaques outside neurons of AD subjects has been shown to stimulate the release of inflammatory mediators by the microglia and astrocytes, thus generating neuroinflammation [46]. Moreover, it has been reported that trimethylamine N-oxide (TNMO), a microbiota-derived metabolite found in higher concentrations in the cerebrospinal fluid of MCI and AD patients, promotes the assembly of hyperphosphorylated tau, a protein that makes up the neurofibrillary tangles inside the neurons of AD subjects [47]. Together, abnormal tau protein and Ab deposits synergistically contribute to neuroinflammation [48]. Through their interaction with TLR-4 receptors in the gut, LPS indirectly activate pro-inflammatory signaling cascades, promoting not only a local inflammatory response and a "leaky gut" but also the onset and progression of low-grade systemic inflammation, further exacerbating neuroinflammation [49].

Conversely, through fermentation, "good" gut bacteria produce anti-inflammatory metabolites, such as short-chain fatty acids (SCFAs). SCFAs can cross the BBB and, through the upregulation of tight junctions, promote its integrity, can modulate LPS-induced microglial inflammatory response, thereby preventing associated neurodegeneration, and can influence the differentiation of immune cells, thus participating in central nervous system immunomodulation [50]. Furthermore, SCFAs have the ability to modulate levels of brain-derived neurotrophic factor (BDNF), which promotes the survival and differentiation of neurons, and have been associated with improved memory function in the cognitively impaired population [51]. Systematically, SCFAs work as "immunomodulators" by influencing cytokines' secretion, which in turn affects the proliferation and differentiation of immune cells. The interaction between SCFAs and the immune system generates anti-inflammatory responses and, at the same time, suppresses LPS-induced pro-inflammatory cytokines such as IL-1β, IL-6, and TNF-α [52]. As such, SCFAs reduce systemic and neural inflammation and help maintain cerebral blood flow and neural circuitry integrity.

Although the composition of a "healthy microbiota" has not been clearly defined, a balanced environment between the host and microorganisms is essential for the maintenance of normal immunological and metabolic functions. Interestingly, analyses of gut microbiota showed differences in composition between healthy, MCI, and AD individuals, with an increase in pro-inflammatory taxa (and associated toxins such as LPS) in those with cognitive impairment [53]. As there is now robust evidence that probiotics not only benefit the host when ingested in sufficient amounts but also may shape the gut microbiota in a way that is neuroprotective, their use in neurocognitive disorders has raised the interest of the scientific community [54-58].

The most prominently studied genera, *Lactobacillus* and *Bifidobacterium*, do not produce LPS and therefore are not pro-inflammatory [59]. Probiotic courses with *Lactobacillus plantarum* and/or *Bifidobacterium breve* lead to improved cognition in AD animal models, which could be explained by their anti-inflammatory and antioxidant properties [60-62]. The efficacy of probiotic supplementation on ameliorating cognitive function in human subjects with some form of cognitive impairment has been reviewed extensively [63-71]. However, most studies combined AD and MCI subjects in their quantitative analyses. Given that evidence suggests that intervention in the MCI stage of AD could improve cognition and delay disease progression, it is imperative to assess MCI subjects independently [64]. One recent review did perform an MCI subgroup analysis, but included trials with questionable eligibility criteria or methodology, potentially devaluating the meta-analysis outcome [71]. By compiling findings of existent primary research, our aim is to determine if probiotic supplementation can improve cognition of those with MCI.

Methods

A targeted literature search was conducted in the PubMed database to identify clinical studies examining the effects of probiotics on cognitive outcomes in patients meeting the clinical criteria for MCI. The search strategy utilized a combination of Medical Subject Headings (MeSH) and keyword terms, including Probiotics [MeSH], Cognitive Dysfunction [MeSH], Alzheimer Disease [MeSH], and related text words (e.g., "mild cognitive impairment", "cognitive impairment", "dementia", and "cognitive behavior*"). Boolean operators were applied to combine search terms and enhance specificity. To capture relevant interventions, the search was limited to studies classified as randomized controlled trials (publication type) or those including terms such as "clinical test" or "randomized controlled trial" in the text. Filters were applied to include only articles available in full text, involving human subjects, published in English, indexed in MEDLINE, and focused on middle-aged and older adults (ages 45 and above). The latest search was conducted on July 15, 2025. 

The following PubMed advanced search query was utilized: Probiotics [mh] OR probiotic* OR probiotics [tiab]) AND (Cognitive Dysfunction [mh] OR "cognitive dysfunction" OR "Mild cognitive impairment" OR "cognitive impairment" OR Alzheimer disease [mh] OR dementia [tw]) AND Treatment outcome [mh] Probiotics [mh] OR probiotic* OR probiotics [tiab]) AND (Cognitive Dysfunction [mh] OR "cognitive dysfunction" OR "Mild cognitive impairment" OR Alzheimer disease [mh] OR "Alzheimer's disease" OR "Alzheimer disease" OR dementia [tw]) AND (clinical trial [pt] OR "clinical test" OR randomized controlled trial [pt]). The filters were as follows: in the last 10 years, Full text, Clinical Trial, Randomized Controlled Trial, English, Humans, MEDLINE.

*Eligibility Criteria* 

Included studies met the following: (1) randomized clinical trial, (2) conducted on humans with objective evidence of MCI, (3) included a probiotic intervention, (4) compared the intervention group with a placebo group, and (4) reported main outcomes of cognitive function using validated neurocognitive function tests. Studies were excluded if (1) the design was unclear or in a language other than English, (2) authors included participants either with intact cognition or with a diagnosis of AD, (3) did not ensure equal distribution of participants' characteristics between study and placebo arms at baseline, (4) administered a prebiotic, or (5) co-supplemented with another micronutrient. 

Data Extraction

**Figure 1:**
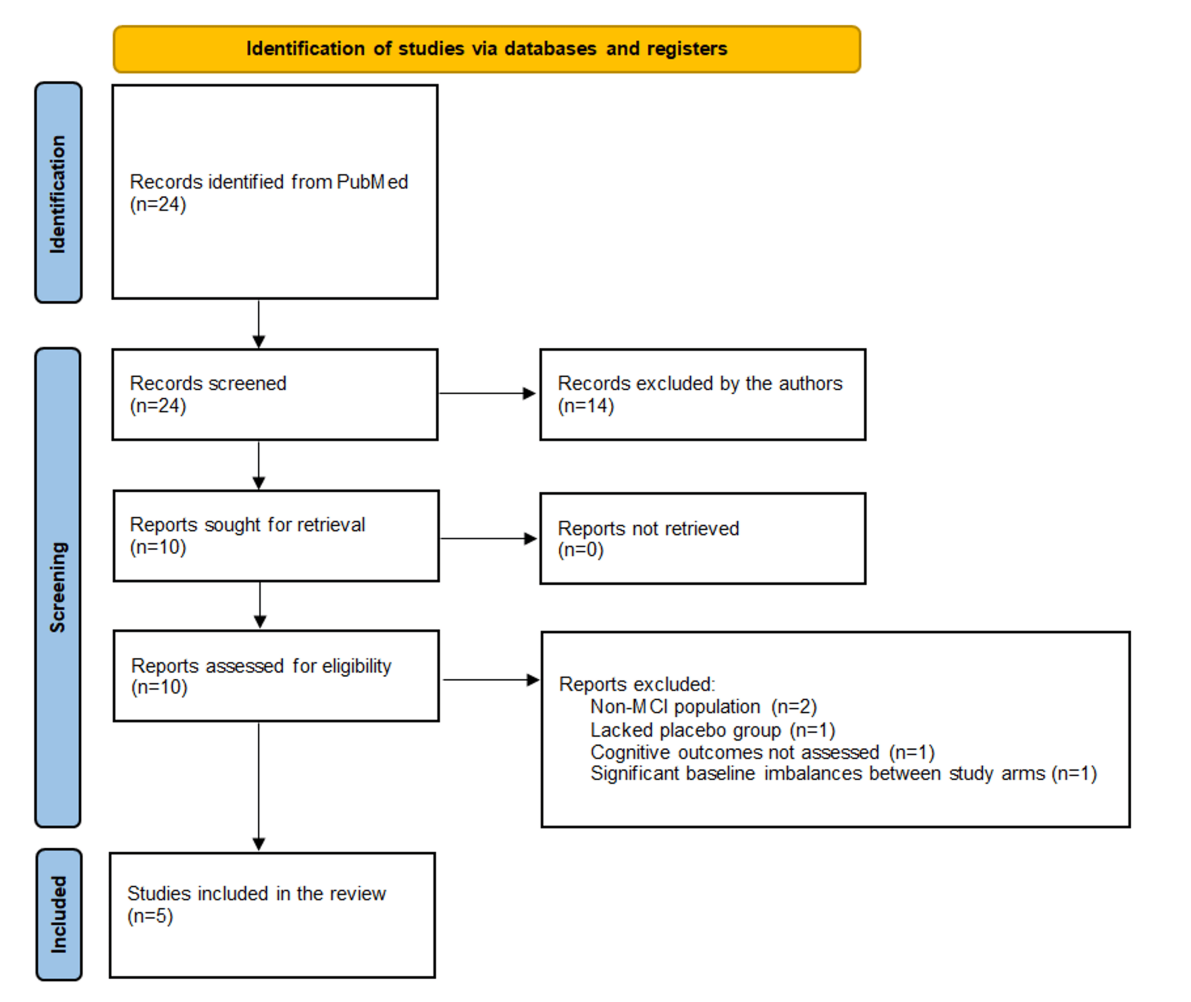
Adapted PRISMA 2020 flow diagram of literature search Flow diagram illustrating the number of studies identified, screened, and considered eligible or not at each point of the review process. PRISMA: Preferred Reporting Items for Systematic Reviews and Meta-Analyses; MCI: mild cognitive impairment

Fourteen studies were excluded based on title or abstract review for the following reasons: the study population had cognitive deficits unrelated to MCI (e.g., chemotherapy-induced, psychiatric), did not meet MCI criteria (e.g., subjective cognitive decline or baseline AD), assessed outcomes unrelated to cognition (e.g., microbiota composition, metabolic or mood changes), focused on a specific clinical context (e.g., pre- vs. postoperative cognition), or used synbiotics rather than probiotics. The remaining 10 full-text articles were reviewed for eligibility. Five were excluded due to non-MCI populations, non-cognitive primary outcomes, lack of a control group, or significant baseline imbalances between study arms. The remaining five met the inclusion criteria and were included in the final review (Figure 1). 

Risk of Bias Assessment

We assessed the methodological quality of all five included randomized controlled trials using the Cochrane Risk of Bias 2 (RoB 2) tool. This tool evaluates five domains: (1) randomization process, (2) deviations from intended interventions, (3) missing outcome data, (4) measurement of the outcome, and (5) selection of the reported result. Each domain was rated as "low risk", "some concerns", or "high risk" of bias. Most trials were rated as low risk across all domains. However, two had some concerns regarding the incomplete reporting of randomization and potential dropout imbalance. A detailed summary of the risk of bias judgments is presented in a table below.

## Review

Results

All studies included were randomized controlled trials conducted in Asia. All studies included were randomized controlled trials conducted in Asia. Participants with objective findings of MCI were included, unless they had evidence of neurodegenerative, brain, or psychiatric disorders, severe metabolic or gut pathologies, irregular lifestyle patterns, or heavy usage of pharmaceutical products such as dietary fibers or probiotics. In each study, participants in both arms were matched at baseline for important characteristics, including age, sex, BMI, and cognitive test performance. Study populations and eligibility criteria are detailed in Table 1.

**Table 1:** Summary of clinical trials evaluating probiotic supplementation in individuals with MCI Study populations consisted of older adults diagnosed with MCI, with study-specific inclusion criteria based on established clinical guidelines or resective cognitive screening thresholds. Key findings reflect between-group differences in cognitive performance (treatment vs. placebo), reported as changes from pretreatment baseline. Outcomes were assessed using validated cognitive measures and are presented using study-defined metrics. RBANS: Repeatable Battery for the Assessment of Neuropsychological Status; MMSE: Mini-Mental State Examination; MCI: mild cognitive impairment; CNT: Computerized Neurocognitive Function Test; ADAS-Jcog: Alzheimer's Disease Assessment Scale-Cognitive Subscale, Japanese version

Study citation	Study population	Therapy of exposure	Key findings
Xiao et al. (2020) [72]	50-79 years old	Daily *B. breve *A1	RBANS score point increment with 95% CI
	MMSE >22	16 weeks	Improved RBANS total score by 11.3 points (6.7-15.8); p<0.0001
	RBANS score correlated with MCI		Improved subdomain "immediate memory" by 9.2 points (5.1-13.3); p<0.0001
			Improved subdomain "delayed memory" by 11.4 points (6.8-16.0); p<0.0001
			Improved subdomain "visuospatial/constructional" by 11.0 points (6.6-15.3); p<0.0001
Asaoka et al. (2022) [73]	65-89 years old	Daily *B. breve *A1	ADAS-Jcog scores
	Met DSM-5 criteria for MCI	24 weeks	Total ADAS-Jcog score: -1.34 (2.61) vs. -1.36 (2.53); p=0.789
			Improved subdomain "orientation" score at week 24: 0.22 (0.67) vs. 0.8 (1.29); p=0.022
			MMSE >25: Improved subdomain "construction" at week 8: 0.45 (0.51) vs. 0.18 (0.39); p=0.021
Kobayashi et al. (2019) [74]	50-80 years old	Daily *B. breve *A1	RBANS score
	MMSE score between 22 and 27	12 weeks	No significant differences in change to RBANS total score: 5.27 +/- 11.25 vs. 4.65 +/- 9.46; p=0.87
			Low score strata: Improved subdomain "immediate memory" score: 3.08 +/- 13.10 vs. -4.22 +/- 11.62; p=0.041
Hwang et al. (2019) [75]	55-85 years old	Daily *L. plantarum *	CNT Z score
	Met DSM-5 criteria for MCI	12 weeks	Improved total CNT combined score at week 12: z=2.36; p=0.02
			Improved subdomain "attention" score: z=2.34; p=0.02
Fei et al. (2023) [76]	>60 years old	Daily strain mixture	MMSE score
	Met Peterson's criteria for MCI	12 weeks	Improved total MMSE score at 12 weeks: 24.75 +/- 2.47 vs. 20.95 +/- 1.36; p<0.001
			Improved subdomain "attention and calculation" score: 3.55 +/- 1.54 vs. 1.60 +/- 0.60; p<0.001
			Improved subdomain "recall" score: 1.95 +/- 0.76 vs. 0.70 +/- 0.47; p<0.001

Xiao et al. [72], Asaoka et al. [73], and Kobayashi et al. [74] used* Bifidobacterium breve* A1 at daily doses of 2.0×10^10^ CFU, with a duration of intervention ranging from 12 to 24 weeks. Using either the Alzheimer's Disease Assessment Scale-Cognitive Subscale, Japanese version (ADAS-Jcog), or the RBANS as primary outcome measures, 1/3 showed statistically significant improvement over placebo in total score following their intervention, while for the other two, significant improvement was observed at the subdomain level, after stratifying for the baseline severity of cognitive impairment. Meanwhile, Hwang et al. [75] used *Lactobacillus plantarum* at daily doses of 1.0×10^10^ CFU for a total of 12 weeks, after which they assessed cognitive progress using three subtests of a CNT battery validated for the MCI population. They reported outcomes as Z scores, which was unique to their study; others reported mean scores. Composite Z scores for each subtest were calculated and averaged to obtain a total score, which was used as their primary outcome measure. The study arm had a significant improvement in total score compared to placebo, following their intervention. Finally, Fei et al. [76] used a commercial mixture containing *Bifidobacterium*, *Lactobacillus*, and *Lactococcus *strains, at daily doses of 4.0×10^10^ CFU for a total of 12 weeks. Cognitive performance was assessed primarily using the MMSE, for which the total score was also significantly improved compared to placebo after the intervention. 

Bifidobacterium breve A1

Asaoka et al. [73] included 115 subjects in their final analysis, of which 55 received a daily sachet containing 2.0×10^10^ CFU of *Bifidobacterium breve* A1 using maize starch as a carrier and 60 received a daily placebo sachet containing maize starch only for 24 weeks. Cognitive performance, based on total ADAS-Jcog and MMSE scores (primary and secondary outcome measures, respectively), was evaluated at baseline and then again at weeks 8, 16, and 24. Changes from baseline to follow-up points were compared between groups, and intragroup changes in values between baseline and the end of the intervention were tested.

ADAS-Jcog and MMSE showed improvement of cognitive function on some subdomain scores, but not in total scores, following the consumption of probiotics by MCI subjects. Those in the probiotic group deteriorated significantly less than those taking the placebo regarding the cognitive domain "orientation" (p=0.021). After stratifying MMSE baseline scores into "more severe" (<25) and "less severe" (≥25), significant score improvements were noted in ADAS-Jcog subdomain "orientation" at 24 weeks (p= 0.022) and for MMSE subdomains "repetition" at week 16 (p=0.0026) and "orientation in time" at week 24 (p=0.006) when compared to placebo.** **In the MMSE >25 strata, no significant intergroup differences were notable. The authors calculated a modified MMSE score (mMMSE) that excluded the Serial-7, based on previous reports indicating better performance of the Japanese population on this part of the test. They also noted a nearly significant improvement of the mMMSE for the full analysis set (p=0.0587) and for the MMSE <25 strata (p=0.061), with no such findings for the MMSE >25 strata. 

Xiao et al. [72] selected 80 subjects for final randomization, of which half (n=40) were randomized to the study arm and received a daily dose equivalent to 2.0×10^10^ CFU of *Bifidobacterium breve *A1 using maize starch as a carrier, while the other half received a daily placebo capsule that contained maize starch only. Participants took the probiotic or placebo capsule for a total of 16 weeks, at which point RBANS and the Japanese version of MCIS (JMCIS) total scores were reassessed (primary and secondary outcome measures, respectively) and the difference in scores between groups was calculated using baseline score as a covariate. One participant was excluded from the probiotic group for consuming prohibited medication. Both intention-to-treat (main) and per-protocol analyses were performed.

In the probiotic group, the RBANS total score showed a significant improvement after 16 weeks of supplementation when compared to placebo (p<0.0001). This significant improvement over placebo was also noted for RBANS subdomain scores in "immediate memory", "delayed memory", and "visuospatial/constructional" (p<0.0001). However, this was not the case for subdomains "attention" and "language", although the ladder showed a trend towards improvement (p=0.064). JMCIS total score also showed significant improvement over placebo (p=0.036) in the per-protocol analysis.

Kobayashi et al. [74] included in their trial 121 participants, of which 61 were randomly allocated to the probiotic group and consumed two capsules of *Bifidobacterium breve *A1 daily (equivalent to >2.0×10^10^ CFU) using maize starch as a carrier, while 60 participants were randomly allocated to the placebo group and consumed two corn starch capsules daily for the duration of the intervention, i.e., 12 weeks. Cognitive performance was assessed at baseline and again at week 12 using RBANS and MMSE scores as primary and secondary outcome measures, respectively. The differences between the *B. breve *A1 and placebo groups were tested, as well as intragroup changes in the values between baseline and 12 weeks.

No significant intergroup difference was noted in terms of changes in total RBANS and MMSE scores at week 12 from those at baseline. Because RBANS scores were significantly different between groups at baseline, a stratified analysis was performed using baseline RBANS "lower score" (<41) and "higher score" (>41). The authors used 41 as a cut-off point because they administered a version of the RBANS for which 41 was established as the limit for a clinical diagnosis of MCI. A significant intergroup difference in changes of scores from baseline of RBANS subdomain "immediate memory" (p=0.041) and total MMSE (p=0.033) was noted in the "lower score" strata at week 12. Moreover, the authors found that the RBANS subdomain score for "delayed memory" was improved from baseline only in the probiotic group (p=0.014). Similarly, MMSE subdomain scores for "orientation in time", "orientation in place", "calculation", and "language" were improved from baseline only in the probiotic group (p=0.018, p=0.027, p=0.044, and p=0.001, respectively). On the other hand, supplementation with *B. breve* A1 showed no notable improvement in the "higher score" strata. Surprisingly, the placebo group showed a greater improvement in total MMSE score compared to the probiotic group in this strata.

Lactobacillus plantarum

Of the 100 patients enrolled in the study of Hwang et al. [75], half (n=50) were randomly assigned to a 0.8 g daily dose of *Lactobacillus plantarum* (equivalent of 1.0×10^10^ CFU) mixed with fermented soybean powder, while the other half received a placebo capsule containing cellulose. The intervention lasted 12 weeks. Ninety-two participants completed the study, with dropout rates similar in both groups. Cognitive performance was measured at baseline and at week 12 using CNT subtests Auditory Continuous Performance Test (ACPT), Digit Span Test (DST), and Verbal Learning Test (VLT), assessing attention, working memory, and verbal memory, respectively. Participants who received the probiotic for 12 weeks showed a statistically significant improvement over placebo in total score (p=0.02). At the subdomain level, a significant improvement in score was noted only for "attention" (p=0.02), although a meaningful trend towards improvement was noted for subdomains related to memory function. 

Mixture of Strains

Fei et al. [76] included 42 participants in their trial, of which half (n=21) were randomly assigned to either the intervention or placebo group. The probiotic group consumed 2 g of commercial probiotic mixture (*Lactobacillus*, *Bifidobacterium*, and *Lactococcus*) equivalent to >4.0×10^10^ CFU and for which the carrier base was not specified. Participants in the placebo group consumed 2 g daily of an unspecified type of starch for the duration of the intervention which was 12 weeks. MMSE and MoCA scores were measured at baseline and again at week 12, as primary and secondary outcome measures, respectively. 

The probiotic group had a higher total MMSE score following the intervention compared to the placebo group, with a significant difference between groups (p<0.001). Additionally, there was a significant intergroup difference between subdomains "attention and calculation" and "recall" (p<0.001). Five patients in the probiotic group improved their MMSE score to 27, indicating rescue of cognitive impairment. However, MMSE subdomains "orientation" and "language" scores were not significantly different between groups. After 12 weeks of intervention, the MoCA total score was higher in those who received the probiotic supplement, compared to placebo (p<0.001). This significant improvement over placebo was also noted for MoCA subdomain scores "visuospatial and executive" and "recall" (p<0.01 and p<0.001, respectively). In sum, they found that the probiotic intervention benefited mostly cognitive domains related to memory and learning abilities.

Risk of Bias Assessment

Risk of bias was evaluated using the Cochrane RoB 2 tool across five domains. Most studies were rated as low risk, with some concerns noted in Asaoka et al. [73] and Fei et al. [76] due to unclear randomization or blinding procedures. A summary is presented in Table 2.

**Table 2:** Risk of bias assessment using the Cochrane Risk of Bias 2 tool MMSE: Mini-Mental State Examination; CNT: Computerized Neurocognitive Function Test; RBANS: Repeatable Battery for the Assessment of Neuropsychological Status

Study	Randomization process	Deviations from intended interventions	Missing outcome data	Measurement of the outcome	Selection of reported result	Overall risk
Xiao et al. (2020) [72]	Low: Randomization and concealment described	Low: Double-blind protocol upheld	Low: Minimal attrition, balanced	Low: Standardized tools, blinded assessors	Low: All outcomes pre-specified	Low
Asaoka et al. (2022) [73]	Some concerns: Randomization described, concealment unclear	Low: Blinded design maintained	Some concerns: Dropout imbalance may affect outcomes	Low: MMSE and CNT used, validated tools	Low: No selective reporting	Some concerns
Kobayashi et al. (2019) [74]	Low: Random permuted blocks, stratified	Low: Blinding of participants and assessors	Low: 3.3% dropout, evenly distributed	Low: RBANS and MMSE, multiple forms to reduce learning bias	Low: All results reported	Low
Hwang et al. (2019) [75]	Low: Randomization and allocation concealment described	Low: Intervention compliance verified	Low: Minimal missing data	Low: Validated tools, assessor-blinded	Low: All data included	Low
Fei et al. (2023) [76]	Some concerns: Randomization not clearly described	Low: Adherence tracked, no protocol deviations	Low: Dropouts acknowledged and balanced	Some concerns: Blinding unclear, possible assessor bias	Low: All cognitive outcomes disclosed	Some concerns

Discussion

The above results are in fact promising with all but Asaoka et al. [73] showing a significant improvement over placebo in total cognitive score, Fei et al. [76] showing a reversal of MCI in five participants, and Xiao et al. [72] showing an 11.3 point increase in total RBANS score over placebo, a remarkable improvement compared to other tested dietary interventions [77,78]. In addition, all showed a statistically significant improvement over placebo in at least one cognitive subdomain, regardless of the neurocognitive test used. Taken together, probiotic interventions benefited all critical domains of MCI, i.e., memory, attention, and executive functions. This may be partly explained by the action of probiotics on the gut-brain axis through the modulation of SCFAs. As discussed earlier, SCFAs cross the BBB and influence synaptic plasticity by upregulating BDNF, enhancing hippocampal neurogenesis, and modulating microglial activity [50-52,60-62]. These mechanisms are increasingly supported by preclinical models and provide a biological rationale for the memory and executive function improvements seen in human trials. Thus, the cognitive benefits observed in these studies may stem not only from gut microbiota rebalancing but also from downstream neurochemical and neurotrophic effects.

Interestingly, there seemed to be a trend of better cognitive performance for those with more severe MCI at baseline. This was made evident by Asaoka et al. [73] as well as Kobayashi et al. [74] and is consistent with findings of previous animal and human trials [79,80]. The exact reason for this phenomenon is unclear, but a possible explanation is that probiotics have limited potential to improve cognition above a certain biologically predetermined set point and consequently may only help those with a clinically evident impairment achieve pre-morbid status. From another angle, we could speculate that probiotics especially benefit those with greater gut microbiota imbalances, such as those with more severe cognitive impairment, compared to less severe cases, which may have no significant imbalance for probiotics to correct, making them less susceptible to this type of intervention, at least with the dosages and durations of intervention tested so far. This being said, MCI is often clinically ignored due to its early symptoms not being obvious. As mentioned earlier, analyses of gut microbial composition revealed distinctions between healthy, MCI, and AD subjects. Since distinguishing age-related cognitive decline and early pathological cognitive decline is a difficult task, gut microbiome analysis may reveal to be a valuable adjunct to neuropsychological screening tests, especially when patients' presentations are sub-clinical, and thus may help identify those susceptible to probiotic interventions before they start performing below expectation on standardized neuropsychological tests, which are known to be insensitive to the subtle deficits of MCI.

While positive overall, some minor discrepancies were noted in the results at the subdomain level. Asaoka et al. [73] showed favorable outcomes mainly in "orientation", while Fei et al. [76] found no such improvement despite also using the MMSE. The latter also failed to do so using the MoCA, despite being more sensitive than the MMSE in the context of MCI [13]. Similarly, both Xiao et al. [72] and Kobayashi et al. [74] showed significant improvement in cognitive domains related to memory function using the RBANS, while Hwang et al. [75] failed to do so using the CNT subtest VLT and DST. Furthermore, both Hwang et al. [75] and Fei et al. [76] showed significant improvement in "attention" using the ACPT and the MMSE, respectively, while Xiao et al. [72] failed to do so using the RBANS, although the baseline score for this subdomain was considerably higher compared to the total score in this study, potentially creating a ceiling effect.

This being said, results were difficult to compare since the tests used, the cognitive subdomains evaluated by each test, the data analyses, and the reporting differed, as illustrated in Table 1. Perhaps, some may have failed to detect differences at the subdomain level due to the lack of sensitivity of the test used. For those who failed to do so using the same test, an equivalent one, or a more sensitive one than a group who did, a possible explanation is that, despite matched intra-study cognitive scores for intervention and placebo groups at baseline, inter-study baseline scores differed greatly for those using the same test. For example, the average baseline MMSE scores spanned the whole MCI spectrum across studies (MMSE 22-26). Additionally, while the average age of participants of Asaoka et al. [73] and Fei et al. [76] was closer to 75, Hwang et al. [75], Xiao et al. [72], and Kobayashi et al. [74] had much younger subjects, together with an average age below 65. Since age and rate of progression of MCI are strongly correlated, and cognitive domains may be affected in a certain order or disproportionately by the degree of impairment, it seems fair to assume that age and baseline cognitive test scores at least partly explain why some studies showed significant improvement in certain subdomains while others failed to do so.

Moreover, the initial composition of the gut microbiome, family history of dementia, education, and many other aspects of demography could have also contributed to the conflicting data at this level. To our knowledge, only two studies considered education level [75,76], while only one considered family history of dementia [76], both of which may influence disease progression, stability or regression over time, and ultimately the outcome of interventions.

Importantly, the size of the study population, dose of probiotic administered, and/or duration of intervention may have been insufficient to detect differences between arms at the subdomain level. Indeed, the study population was small (42-130 subjects), limiting study power; daily dosage of probiotic ranged from 2.0 to 4.0×10^10^ CFU, which could have influenced outcomes differently if a dose-dependent relationship exists; the shortest trial was 12 weeks, and although 12 weeks is largely implemented to observe the effects of dietary supplements on cognition, it may be not be true at the subdomain level [81-83]. In addition, the choice of bacterial strain, the variable quality that exists between the same strains, and a single- versus multi-strain intervention may have participated in conflicting results, not to mention that the type of carrier used may have acted as a synbiotic, such as in the case of the fermented soybean powder used by Hwang et al. [75], possibly also explaining why the authors obtained results similar to that of other trials despite using smaller doses.

Limitations

Despite promising, these findings should be interpreted with caution. Asaoka et al. [73] needed a minimum of 70 participants in each group to be sufficiently powered, which they were unable to reach due to dropouts, which favored the placebo group; Hwang et al. [75] did not evaluate all critical subdomains of MCI; Kobayashi et al. [74] reported a flawed study design with RBANS scores differing significantly between study and placebo arms at baseline. Furthermore, the choice of diagnostic criteria and neuropsychological tests used to assess outcomes varied across studies, and not all tests were validated in the context of MCI (e.g., MMSE) [84]. Importantly, proper follow-ups would have allowed the authors to assess whether cognitive improvement was maintained over time, a relevant aspect of the intervention considering the chronic and progressive nature of the disease. While most included trials were assessed as low risk using the Cochrane RoB 2 tool, some concerns related to randomization and outcome blinding were noted in two studies, which may have introduced bias into individual findings.

The biggest limitation was the paucity of clinical trials, in that five studies met our inclusion criteria, for a total study population under 500 individuals, although one could argue that we could have included two additional randomized controlled trials [85,86]. However, certain aspects of their methodology made them ineligible. In the case of Sanborn et al. [85], BMI was unmatched between arms at baseline, introducing a potential confounder [87]. Similarly, subjects with depression should have been excluded, being a well-recognized medical cause of cognitive decline [88]. On the other hand, Sakurai et al. [86] set their inclusion criteria so that those with an MCIS Memory Performance Index score of <60 would be candidates, although the cut-off for MCI using this scale, according to Rafii et al. [18], is <50. Participants' performance on cognitive subtests of a CNT battery administered at baseline was suggestive of very early memory deficits, rather than overt MCI, some of which could have been attributed to other medical conditions affecting memory, perhaps mood or metabolic disorders, which did not specifically meet the exclusion criteria. Nevertheless, findings were positive in both these randomized controlled trials, corroborating those presented in our review. Coming back to our own limitations, all randomized controlled trials were conducted in Asia, which limits the generalizability of findings quite substantially. Indeed, ethnicity, such as being Asian in this case, may influence probiotic efficacy due to genetic differences in gut microbiota composition and immune responses. Additionally, traditional Asian diets rich in fermented foods (e.g., kimchi, miso) may precondition the gut environment, potentially enhancing or altering the effects of supplemental probiotics.

Probiotics *Lactobacillus *and *Bifidobacterium *are known to be safe and well-tolerated, with no absolute contraindication or known drug interactions [89]. In fact, no significant side effects from probiotic intake were reported in the trials included in this review. This is important, considering the higher prevalence of comorbidities and polypharmacy among the age group targeted for MCI intervention. However, our objective was to assess the benefits of probiotic intervention on cognition of otherwise healthy MCI subjects, i.e., current evidence cannot be generalized to those with active serious illnesses, other neurocognitive disorders, or obvious brain or gut pathologies. Moreover, many probiotics are sold as dietary supplements, i.e., do not require FDA approval before they can be marketed. Thus, the quality of commercially available probiotics may not be equivalent to that of clinical trials. Consequently, the benefits seen in the research setting cannot be extrapolated to the clinical setting at this point. If a probiotic is going to be marketed as a drug for the treatment of MCI, it will have to meet stricter FDA requirements [90].

Future Directions

To better assess the clinical relevance of probiotic interventions in MCI, future research should include longer-term randomized controlled trials, ideally extending to 12 months or more, to determine whether cognitive benefits are durable and translate into delayed progression to dementia. Head-to-head comparisons between single-strain and multi-strain formulations are also warranted to evaluate whether combining species yields additive, synergistic, or potentially antagonistic effects. In parallel, integrating gut microbiome profiling into study protocols could help identify microbial signatures predictive of treatment response, advancing the field towards more personalized and effective probiotic strategies.

Additionally, larger-scale multicenter clinical trials that adhere to the gold-standard diagnostic criteria for MCI such as the Petersen criteria, often refined in clinical and research contexts by the National Institute on Aging-Alzheimer's Association, are also required to confirm present findings and assess their clinical relevance. These refinements would not only enhance reproducibility and mechanistic insight but also support the development of evidence-based probiotic interventions for MCI.

## Conclusions

The growing prevalence of neurodegenerative diseases among aging populations underscores the urgent need for early, preventive treatment strategies that can interrupt or delay cognitive decline. Central to many of these disorders, including MCI, Lewy body dementia, and AD, is a chronic neuroinflammatory process driven by both local and systemic factors. Disruption of the BBB and prolonged glial activation contribute to irreversible damage that, once established, cannot be reversed by current therapies. As such, targeting upstream drivers of neuroinflammation with probiotics may offer a more effective means of preserving cognitive function before neurodegeneration becomes advanced.

Emerging evidence implicates the gut microbiome as a key modulator of neuroinflammation through the production of pro- and anti-inflammatory metabolites. Probiotic supplementation with anti-inflammatory strains such as *Lactobacillus* and *Bifidobacterium* has shown promising results in patients with MCI, improving cognitive performance across memory, attention, and executive domains. In contrast to the mixed outcomes of trials combining MCI and AD populations, the present review highlights consistent benefits when probiotics are evaluated solely in individuals with MCI, suggesting a therapeutic window where intervention may be most effective. These findings support the potential of probiotic therapy as a low-risk, cost-effective strategy to mitigate neuroinflammation, delay cognitive decline, and reduce the incidence of neurodegenerative disease in aging individuals. As the body of evidence grows, probiotic supplementation may emerge as a practical adjunct to early intervention efforts aimed at preserving cognition in the aging brain.
